# 4-Phenyl-1,2,3,4-tetra­hydro­pyrimido[1,2-*a*]benzimidazol-2-one

**DOI:** 10.1107/S1600536808033497

**Published:** 2008-10-18

**Authors:** Gong-Chun Li, Feng-Ling Yang, Chang-Sheng Yao

**Affiliations:** a College of Chemistry and Chemical Engineering, Xuchang University, Xuchang, Henan Province 461000, People’s Republic of China; b School of Chemistry and Chemical Engineering, Xuzhou Normal University, Xuzhou 221116, People’s Republic of China, and Key Laboratory of Biotechnology for Medicinal Plants, Xuzhou Normal University, Xuzhou 221116, People’s Republic of China

## Abstract

In the title compound, C_16_H_13_N_3_O, the tetrahydropyrimidin­one ring adopts a sofa conformation. In the crystal structure, mol­ecules are linked by N—H⋯N hydrogen bonds and C—H⋯π inter­actions.

## Related literature

For background information on the biological activities of derivatives of benzo[4,5]imidazo[1,2-*a*]pyrimidine, see: Abdel-Hafez (2007 ▶); Cheung *et al.* (2002 ▶); Nunes, Zhu, Amouzegh *et al.* (2005 ▶); Nunes, Zhu, Ermann *et al.* (2005 ▶).
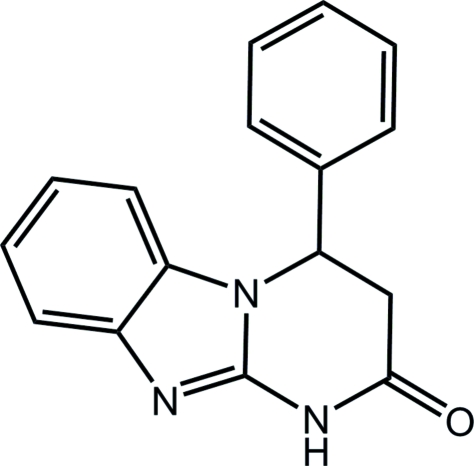


## Experimental

### 

#### Crystal data

C_16_H_13_N_3_O
*M* *_r_* = 263.29
Orthorhombic, 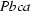

*a* = 13.606 (3) Å
*b* = 7.5674 (15) Å
*c* = 24.578 (5) Å
*V* = 2530.6 (9) Å^3^
*Z* = 8
Mo *K*α radiation
μ = 0.09 mm^−1^
*T* = 113 (2) K
0.18 × 0.16 × 0.12 mm

#### Data collection

Rigaku Saturn diffractometer
Absorption correction: multi-scan (*CrystalClear*; Rigaku/MSC, 2002 ▶) *T*_min_ = 0.984, *T*_max_ = 0.989
18521 measured reflections
2232 independent reflections
2075 reflections with *I* > 2σ(*I*)
*R*_int_ = 0.035

#### Refinement

*R*[*F* ^2^ > 2σ(*F* ^2^)] = 0.037
*wR*(*F* ^2^) = 0.112
*S* = 1.15
2232 reflections
185 parameters
1 restraint
H atoms treated by a mixture of independent and constrained refinement
Δρ_max_ = 0.21 e Å^−3^
Δρ_min_ = −0.28 e Å^−3^

### 

Data collection: *CrystalClear* (Rigaku/MSC, 2002 ▶); cell refinement: *CrystalClear*; data reduction: *CrystalClear*; program(s) used to solve structure: *SHELXS97* (Sheldrick, 2008 ▶); program(s) used to refine structure: *SHELXL97* (Sheldrick, 2008 ▶); molecular graphics: *SHELXTL* (Sheldrick, 2008 ▶); software used to prepare material for publication: *SHELXTL*.

## Supplementary Material

Crystal structure: contains datablocks I, global. DOI: 10.1107/S1600536808033497/bt2811sup1.cif

Structure factors: contains datablocks I. DOI: 10.1107/S1600536808033497/bt2811Isup2.hkl

Additional supplementary materials:  crystallographic information; 3D view; checkCIF report

## Figures and Tables

**Table 1:** Hydrogen-bond geometry (Å, °) *Cg* is the centroid of the C11–C16 ring.

*D*—H⋯*A*	*D*—H	H⋯*A*	*D*⋯*A*	*D*—H⋯*A*
N1—H1⋯N3^i^	0.901 (9)	1.909 (10)	2.8027 (17)	171.0 (19)
C13—H13⋯*Cg*^ii^	0.93	2.85	3.6296 (18)	143

Symmetry codes: (i) 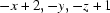; (ii) 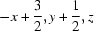.

**Fig. 1.**
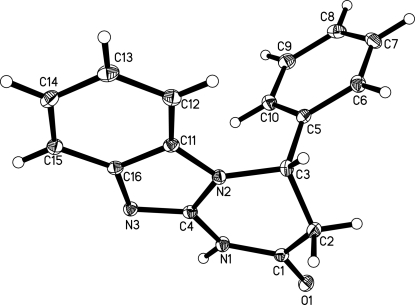
The molecular structure of the title compound, showing 30% probability displacement ellipsoids and the atom-numbering scheme.

**Fig. 2.**
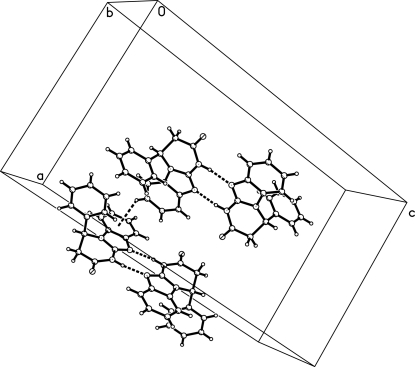
The packing diagram of the title compound. Intermolecular hydrogen bonds are shown as dashed lines.

## supplementary crystallographic information

### Comment

Among the derivatives of dihydropyrimidine, the derivatives of benzo[4,5]imidazo[1,2-*a*]-pyrimidine have been reported to have a variety of biological activities, such as antineoplastic activity (Abdel-Hafez, 2007), protein kinase inhibitor (Nunes, Zhu, Amouzegh *et al.*, 2005), T cell activation (Nunes, Zhu, Ermann *et al.*, 2005), TIE-2 and/or VEGFR2 inhibitory activities (Cheung *et al.*, 2002). This led us to pay much attention to the synthesis and bioactivity of these important fused heterocyclic compounds. To further study the relationship between structure and bioactivity, we synthesised a series of derivatives of benzo[4,5]imidazo[1,2-*a*]-pyrimidine. Here we report the crystal structure of the title compound.

In the title molecule (Fig.1), the pyrimidine ring adopts a sofa conformation. The phenyl ring is almost perpendicular to the pyrimidine plane [dihedral angle 89.00 (3)°].

The crystal packing is stabilized by an N—H···N   hydrogen bond, and a C—H···π interaction (Table 1, Fig. 2).

### Experimental

The title compound was synthesized by the reaction of benzaldehyde (1 mmol), 2,2-dimethyl-1,3-dioxane-4,6-dione (1 mmol) and 1*H*-benzo[*d*]imidazol-2-amine (1 mmol) in 3-butyl-1-methyl-1H-imidazol-3-ium chloride (1.5 mL) at 363 K for a certain time (monitered by TLC). After cooling, the reaction mixture was washed with water and recrystallized from ethanol, to obtain single crystals suitable for X-ray diffraction.

### Refinement

The hydrogen atom bonded to the nitrogen atom was located in a Fourier difference map and was refined with a distance restraint of 0.90 Å with an estimated standard deviation of 0.01 Å. Other H atoms were placed in calculated positions (C—H = 0.93–0.98 Å) and included in the final cycles of refinement using a riding model, with *U*_iso_(H) = 1.2*U*_eq_(C).

### Crystal data

**Table:**

C_16_H_13_N_3_O	*D*_x_ = 1.382 Mg m^−^^3^
*M**_r_* = 263.29	Mo *K*α radiation, λ = 0.71073 Å
Orthorhombic, *P**b**c**a*	Cell parameters from 5932 reflections
*a* = 13.606 (3) Å	θ = 1.5–27.9°
*b* = 7.5674 (15) Å	µ = 0.09 mm^−^^1^
*c* = 24.578 (5) Å	*T* = 113 K
*V* = 2530.6 (9) Å^3^	Block, colourless
*Z* = 8	0.18 × 0.16 × 0.12 mm
*F*(000) = 1104	

### Data collection

**Table:**

Rigaku Saturn diffractometer	2232 independent reflections
Radiation source: rotating anode	2075 reflections with *I* > 2σ(*I*)
confocal	*R*_int_ = 0.035
ω scans	θ_max_ = 25.0°, θ_min_ = 1.7°
Absorption correction: multi-scan (CrystalClear; Rigaku/MSC, 2002)	*h* = −14→16
*T*_min_ = 0.984, *T*_max_ = 0.989	*k* = −9→9
18521 measured reflections	*l* = −29→29

### Refinement

**Table:**

Refinement on *F*^2^	Primary atom site location: structure-invariant direct methods
Least-squares matrix: full	Secondary atom site location: difference Fourier map
*R*[*F*^2^ > 2σ(*F*^2^)] = 0.037	Hydrogen site location: inferred from neighbouring sites
*wR*(*F*^2^) = 0.112	H atoms treated by a mixture of independent and constrained refinement
*S* = 1.15	*w* = 1/[σ^2^(*F*_o_^2^) + (0.0639*P*)^2^ + 0.7741*P*] where *P* = (*F*_o_^2^ + 2*F*_c_^2^)/3
2232 reflections	(Δ/σ)_max_ < 0.001
185 parameters	Δρ_max_ = 0.21 e Å^−^^3^
1 restraint	Δρ_min_ = −0.28 e Å^−^^3^

### Special details

**Table:**

Geometry. All esds (except the esd in the dihedral angle between two l.s. planes) are estimated using the full covariance matrix. The cell esds are taken into account individually in the estimation of esds in distances, angles and torsion angles; correlations between esds in cell parameters are only used when they are defined by crystal symmetry. An approximate (isotropic) treatment of cell esds is used for estimating esds involving l.s. planes.
Refinement. Refinement of F^2^ against ALL reflections. The weighted R-factor wR and goodness of fit S are based on F^2^, conventional R-factors R are based on F, with F set to zero for negative F^2^. The threshold expression of F^2^ > 2sigma(F^2^) is used only for calculating R-factors(gt) etc. and is not relevant to the choice of reflections for refinement. R-factors based on F^2^ are statistically about twice as large as those based on F, and R- factors based on ALL data will be even larger.

### Fractional atomic coordinates and isotropic or equivalent isotropic displacement parameters (Å^2^)

**Table:**

	*x*	*y*	*z*	*U*_iso_*/*U*_eq_	
O1	1.17082 (8)	−0.08466 (14)	0.37667 (4)	0.0259 (3)	
N1	1.05925 (9)	0.04671 (16)	0.43091 (5)	0.0194 (3)	
N2	0.98285 (9)	0.31430 (15)	0.40471 (4)	0.0169 (3)	
N3	0.95726 (9)	0.21752 (16)	0.48971 (5)	0.0174 (3)	
C1	1.11740 (10)	0.0412 (2)	0.38584 (6)	0.0197 (3)	
C2	1.11420 (10)	0.2022 (2)	0.34972 (6)	0.0207 (3)	
H2A	1.1314	0.1673	0.3130	0.025*	
H2B	1.1635	0.2856	0.3621	0.025*	
C3	1.01412 (10)	0.29573 (19)	0.34843 (5)	0.0183 (3)	
H3	1.0234	0.4141	0.3331	0.022*	
C4	1.00072 (10)	0.18918 (18)	0.44299 (5)	0.0168 (3)	
C5	0.93882 (10)	0.19885 (19)	0.31400 (6)	0.0187 (3)	
C6	0.93352 (11)	0.2371 (2)	0.25863 (6)	0.0225 (4)	
H6	0.9748	0.3225	0.2438	0.027*	
C7	0.86734 (12)	0.1491 (2)	0.22546 (6)	0.0257 (4)	
H7	0.8639	0.1764	0.1886	0.031*	
C8	0.80641 (11)	0.0207 (2)	0.24712 (6)	0.0261 (4)	
H8	0.7624	−0.0392	0.2248	0.031*	
C9	0.81109 (11)	−0.0182 (2)	0.30193 (6)	0.0259 (4)	
H9	0.7702	−0.1045	0.3165	0.031*	
C10	0.87656 (11)	0.0711 (2)	0.33525 (6)	0.0229 (4)	
H10	0.8788	0.0451	0.3722	0.028*	
C11	0.92020 (10)	0.43649 (18)	0.42860 (6)	0.0169 (3)	
C12	0.87700 (10)	0.58958 (19)	0.40932 (6)	0.0211 (3)	
H12	0.8868	0.6287	0.3739	0.025*	
C13	0.81828 (11)	0.6814 (2)	0.44562 (6)	0.0239 (4)	
H13	0.7873	0.7846	0.4343	0.029*	
C14	0.80457 (11)	0.6224 (2)	0.49899 (6)	0.0220 (4)	
H14	0.7646	0.6873	0.5223	0.026*	
C15	0.84877 (10)	0.47015 (19)	0.51797 (6)	0.0189 (3)	
H15	0.8403	0.4329	0.5537	0.023*	
C16	0.90620 (10)	0.37505 (19)	0.48182 (5)	0.0164 (3)	
H1	1.0597 (14)	−0.043 (2)	0.4548 (6)	0.040 (5)*	

### Atomic displacement parameters (Å^2^)

**Table:**

	*U*^11^	*U*^22^	*U*^33^	*U*^12^	*U*^13^	*U*^23^
O1	0.0249 (6)	0.0282 (6)	0.0246 (6)	0.0092 (5)	0.0023 (4)	−0.0037 (5)
N1	0.0215 (7)	0.0183 (7)	0.0185 (6)	0.0044 (5)	0.0020 (5)	0.0009 (5)
N2	0.0180 (6)	0.0174 (6)	0.0154 (6)	0.0013 (5)	0.0003 (5)	−0.0006 (5)
N3	0.0171 (6)	0.0169 (7)	0.0182 (6)	0.0007 (5)	0.0000 (5)	−0.0007 (5)
C1	0.0158 (7)	0.0241 (8)	0.0192 (7)	0.0005 (6)	−0.0014 (6)	−0.0036 (6)
C2	0.0171 (7)	0.0247 (8)	0.0202 (7)	−0.0016 (6)	0.0021 (6)	−0.0015 (6)
C3	0.0197 (7)	0.0190 (8)	0.0161 (7)	−0.0006 (6)	0.0030 (5)	0.0007 (6)
C4	0.0151 (7)	0.0169 (7)	0.0184 (7)	−0.0004 (5)	−0.0015 (5)	−0.0009 (5)
C5	0.0174 (7)	0.0192 (8)	0.0195 (7)	0.0041 (5)	0.0010 (6)	−0.0009 (6)
C6	0.0263 (8)	0.0209 (8)	0.0204 (7)	0.0035 (6)	0.0023 (6)	0.0024 (6)
C7	0.0313 (9)	0.0273 (8)	0.0186 (7)	0.0087 (7)	−0.0048 (6)	−0.0012 (6)
C8	0.0240 (8)	0.0237 (8)	0.0306 (8)	0.0057 (6)	−0.0082 (6)	−0.0069 (7)
C9	0.0220 (8)	0.0252 (8)	0.0303 (8)	−0.0023 (6)	−0.0007 (6)	−0.0012 (6)
C10	0.0225 (8)	0.0264 (8)	0.0199 (7)	−0.0009 (6)	−0.0001 (6)	0.0022 (6)
C11	0.0136 (7)	0.0170 (7)	0.0200 (7)	−0.0018 (5)	−0.0016 (5)	−0.0024 (6)
C12	0.0209 (8)	0.0199 (8)	0.0224 (7)	−0.0003 (6)	−0.0022 (6)	0.0021 (6)
C13	0.0221 (8)	0.0179 (8)	0.0316 (8)	0.0039 (6)	−0.0032 (6)	0.0009 (6)
C14	0.0169 (7)	0.0200 (8)	0.0291 (8)	0.0014 (6)	0.0007 (6)	−0.0054 (6)
C15	0.0162 (7)	0.0199 (8)	0.0208 (7)	−0.0029 (6)	0.0000 (6)	−0.0025 (6)
C16	0.0141 (7)	0.0154 (7)	0.0197 (7)	−0.0019 (5)	−0.0019 (5)	−0.0007 (5)

### Geometric parameters (Å, °)

**Table:**

O1—C1	1.2192 (18)	C6—H6	0.9300
N1—C1	1.3619 (18)	C7—C8	1.384 (2)
N1—C4	1.3728 (18)	C7—H7	0.9300
N1—H1	0.901 (9)	C8—C9	1.380 (2)
N2—C4	1.3568 (18)	C8—H8	0.9300
N2—C11	1.3879 (18)	C9—C10	1.386 (2)
N2—C3	1.4541 (17)	C9—H9	0.9300
N3—C4	1.3091 (18)	C10—H10	0.9300
N3—C16	1.3933 (19)	C11—C12	1.383 (2)
C1—C2	1.508 (2)	C11—C16	1.4013 (19)
C2—C3	1.535 (2)	C12—C13	1.385 (2)
C2—H2A	0.9700	C12—H12	0.9300
C2—H2B	0.9700	C13—C14	1.398 (2)
C3—C5	1.518 (2)	C13—H13	0.9300
C3—H3	0.9800	C14—C15	1.381 (2)
C5—C10	1.388 (2)	C14—H14	0.9300
C5—C6	1.393 (2)	C15—C16	1.385 (2)
C6—C7	1.385 (2)	C15—H15	0.9300
			
C1—N1—C4	122.48 (12)	C8—C7—C6	120.03 (14)
C1—N1—H1	120.3 (13)	C8—C7—H7	120.0
C4—N1—H1	117.2 (13)	C6—C7—H7	120.0
C4—N2—C11	106.36 (11)	C9—C8—C7	119.84 (14)
C4—N2—C3	122.67 (12)	C9—C8—H8	120.1
C11—N2—C3	130.28 (12)	C7—C8—H8	120.1
C4—N3—C16	104.09 (11)	C8—C9—C10	120.16 (15)
O1—C1—N1	121.37 (14)	C8—C9—H9	119.9
O1—C1—C2	122.66 (13)	C10—C9—H9	119.9
N1—C1—C2	115.93 (12)	C9—C10—C5	120.64 (14)
C1—C2—C3	114.22 (12)	C9—C10—H10	119.7
C1—C2—H2A	108.7	C5—C10—H10	119.7
C3—C2—H2A	108.7	C12—C11—N2	132.40 (13)
C1—C2—H2B	108.7	C12—C11—C16	122.69 (13)
C3—C2—H2B	108.7	N2—C11—C16	104.91 (12)
H2A—C2—H2B	107.6	C11—C12—C13	116.43 (14)
N2—C3—C5	112.30 (11)	C11—C12—H12	121.8
N2—C3—C2	106.53 (11)	C13—C12—H12	121.8
C5—C3—C2	112.82 (12)	C12—C13—C14	121.39 (14)
N2—C3—H3	108.3	C12—C13—H13	119.3
C5—C3—H3	108.3	C14—C13—H13	119.3
C2—C3—H3	108.3	C15—C14—C13	121.71 (14)
N3—C4—N2	114.39 (12)	C15—C14—H14	119.1
N3—C4—N1	125.47 (13)	C13—C14—H14	119.1
N2—C4—N1	120.13 (12)	C14—C15—C16	117.56 (13)
C10—C5—C6	118.75 (13)	C14—C15—H15	121.2
C10—C5—C3	122.61 (13)	C16—C15—H15	121.2
C6—C5—C3	118.62 (13)	C15—C16—N3	129.56 (13)
C7—C6—C5	120.56 (14)	C15—C16—C11	120.20 (13)
C7—C6—H6	119.7	N3—C16—C11	110.24 (12)
C5—C6—H6	119.7		
			
C4—N1—C1—O1	177.97 (13)	C5—C6—C7—C8	−0.6 (2)
C4—N1—C1—C2	0.3 (2)	C6—C7—C8—C9	0.6 (2)
O1—C1—C2—C3	149.84 (14)	C7—C8—C9—C10	0.0 (2)
N1—C1—C2—C3	−32.47 (18)	C8—C9—C10—C5	−0.7 (2)
C4—N2—C3—C5	87.47 (16)	C6—C5—C10—C9	0.7 (2)
C11—N2—C3—C5	−81.67 (17)	C3—C5—C10—C9	−177.88 (14)
C4—N2—C3—C2	−36.51 (17)	C4—N2—C11—C12	−179.39 (15)
C11—N2—C3—C2	154.34 (14)	C3—N2—C11—C12	−8.9 (2)
C1—C2—C3—N2	47.56 (15)	C4—N2—C11—C16	1.18 (15)
C1—C2—C3—C5	−76.11 (15)	C3—N2—C11—C16	171.67 (13)
C16—N3—C4—N2	−0.06 (16)	N2—C11—C12—C13	−179.24 (14)
C16—N3—C4—N1	−179.26 (13)	C16—C11—C12—C13	0.1 (2)
C11—N2—C4—N3	−0.74 (16)	C11—C12—C13—C14	0.6 (2)
C3—N2—C4—N3	−172.13 (12)	C12—C13—C14—C15	0.0 (2)
C11—N2—C4—N1	178.50 (12)	C13—C14—C15—C16	−1.2 (2)
C3—N2—C4—N1	7.1 (2)	C14—C15—C16—N3	−178.84 (13)
C1—N1—C4—N3	−166.90 (14)	C14—C15—C16—C11	1.8 (2)
C1—N1—C4—N2	13.9 (2)	C4—N3—C16—C15	−178.53 (14)
N2—C3—C5—C10	−30.14 (19)	C4—N3—C16—C11	0.85 (15)
C2—C3—C5—C10	90.27 (16)	C12—C11—C16—C15	−1.3 (2)
N2—C3—C5—C6	151.27 (13)	N2—C11—C16—C15	178.17 (12)
C2—C3—C5—C6	−88.32 (16)	C12—C11—C16—N3	179.23 (12)
C10—C5—C6—C7	0.0 (2)	N2—C11—C16—N3	−1.28 (15)
C3—C5—C6—C7	178.60 (13)		

### Hydrogen-bond geometry (Å, °)

**Table:**

*D*—H···*A*	*D*—H	H···*A*	*D*···*A*	*D*—H···*A*
N1—H1···N3^i^	0.90 (1)	1.91 (1)	2.8027 (17)	171 (2)
C13—H13···Cg^ii^	0.93	2.85	3.6296 (18)	143

Symmetry codes: (i) −*x*+2, −*y*, −*z*+1; (ii) −*x*+3/2, *y*+1/2, *z*.
